# Prevalence and predictors for periodontitis among adults in China, 2010

**DOI:** 10.3402/gha.v7.24503

**Published:** 2014-07-08

**Authors:** Qi Zhang, Zhixin Li, Chunxiao Wang, Tao Shen, Yang Yang, Saipin Chotivichien, Linhong Wang

**Affiliations:** 1 National Center for Chronic and Non-communicable Disease Control and Prevention, Chinese Center for Disease Control and Prevention, Beijing, China; 2 Chinese Field Epidemiology Training Program (CFETP), Chinese Center for Disease Control and Prevention, Beijing, China; 3 Bureau of Nutrition, Department of Health, Ministry of Public Health, Nonthaburi, Thailand

**Keywords:** periodontitis, predictors, prevalence, adults

## Abstract

**Background:**

Although the interrelationship between poor oral health and chronic diseases is well established, few related studies are available in China. In this study, the prevalence of severe periodontitis and its association with chronic diseases among adults in China have been explored.

**Design:**

During China's 2010 Chronic Disease and Risk Factor Surveillance (CCDRFS) survey among adults aged 18 and older, 3 out of 162 surveillance points and the entire sample from each point (600×3=1,800 subjects) were selected as pilot study sites in which oral examination was performed. Basic demographic information, chronic diseases status, and results of oral examination were collected from 2010 CCDRFS data. A standard oral examination was conducted by trained staff. Periodontitis was defined as moderate (4–5 mm pockets) or severe (≥6 mm pockets). Chronic disease status was determined by using standard methods and criteria. Multivariate logistic analysis was used to identify the independent association of various factors with severe periodontitis.

**Results:**

Of 1,800 subjects, 1,728 subjects (96%) provided complete information. The prevalence of severe periodontitis was 1.9% (32/1,728) (95% CI=1.2–2.5). In multivariate model, participants with diabetes were 2.4 times (OR=2.4, 95% CI=1.1–5.6) more likely to have severe periodontitis. Being male was significantly associated with severe periodontitis (OR=3.5, 95% CI=1.6–7.7). Living in a rural area was related to an increased chance of having severe periodontitis (OR=2.1, 95% CI=1.0– 4.9). Attainment of at least 6 years of education was inversely associated with severe periodontitis (OR=0.3, 95% CI=0.1–0.8).

**Conclusions:**

According to this pilot project, prevalence of severe periodontitis was low. Control measures should be particularly emphasized for high-risk groups such as less educated people (<6 years of education), people living in rural areas, men, and diabetes patients. Population-based studies, including oral examination by trained staff, are feasible and should be done in order to understand the burden of periodontitis and to provide an effective response to this key oral health issue.

Oral health is vital to the general health and well-being of all populations. The mouth reflects a person's health and well-being throughout life. Oral disease can have an impact on many aspects of general health, and health conditions can in turn have an impact on oral health. Prevalence of oral disease and chronic diseases such as diabetes mellitus (DM), hypertension, and hyperlipidemia increases with age; therefore, it is important to examine the interplay of chronic diseases with oral disease. Globally, periodontitis and caries are among the most prevalent oral diseases. Also, periodontitis accounts for most cases of tooth loss, and their impact increases with age. Periodontal diseases, including gingivitis, gum bleeding, calculus, and periodontitis, are prevalent among the Chinese. Currently, in China, Europe, and the United States, periodontitis is reported to have affected more than half of the adult population (1–4). In ageing populations, the prevalence of periodontitis is even higher. It is reported that 70–90% of individuals aged between 60 and 74 suffer from periodontal disease (1, 4, 5).

Periodontitis is associated with smoking, inadequate oral hygiene, diabetes, hypertension, rheumatoid arthritis, depression, anxiety, obesity, and other risk factors, including nutrition, alcohol consumption, socioeconomic status, and stress (6). In Western countries, many studies have examined the relationship between periodontitis and chronic diseases. Periodontitis has an impact on diabetes, cardiovascular diseases, and adverse pregnancy outcomes (7–9). Moreover, recently there is much emphasis on the ‘two-way’ relationship between diabetes and periodontitis (10).

Despite numerous studies of the association between oral disease and chronic diseases, these studies have not systematically explored the relationship between periodontitis and the socio-demographic determinants and the health conditions of the Chinese. China's 2010 Chronic Disease and Risk Factor Surveillance (CCDRFS) survey is an ongoing, nationally representative surveillance survey administered by China's National Center for Chronic and Non-communicable Disease Control and Prevention. The 2010 CCDRFS was carried out from August to November 2010 using the national disease surveillance points system, which encompassed 162 districts/counties and all 31 provinces, autonomous regions, and municipalities in mainland China. The CCDRFS collected population-based information on many health domains, including sociodemographics, health behaviors, health conditions, disabilities, health care access, self-reported oral health behaviors and oral conditions through oral examination by trained staff (only in three pilot surveillance points).

This study investigated useful strategies to estimate the burden of periodontitis among adults in China. Its specific objective was to identify the prevalence and predictors of severe periodontitis and the association between periodontitis and determinants of demographic information, including gender, education level, and chronic diseases by using data from 2010 CCDRFS survey.

## Methods

### Subjects and sampling

The 2010 CCDRFS was conducted by gathering participants aged more than 18 in certain central locations. The procedure of multistage stratified cluster sampling and the collection of data on behavioral risk factors for non-communicable diseases and physical examination have been published elsewhere (11–14). The detailed description of measurement of biological indicators and data analysis were as used in recent published literature (12). Briefly, in the first stage of sampling, four townships were randomly selected from each surveillance district/county using the method of probability proportional to size. Three villages or residential areas were then selected from each chosen township by using the same method as in the previous stage. Subsequently, a residential group (at least 50 families) was selected from each chosen village or residential area by simple random sampling. Finally, an individual aged at least 18 was selected in each family by means of a Kish grid. Six hundred subjects were enrolled in each surveillance district/county.

**Fig. 1:**
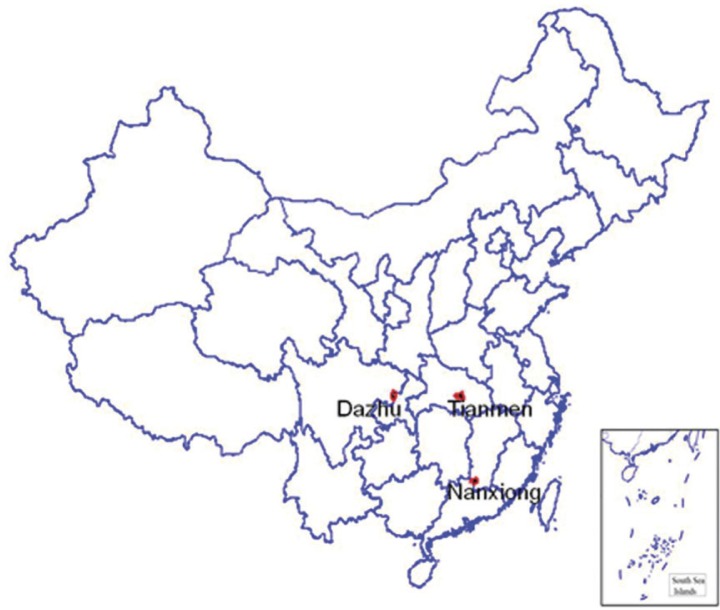
The location of three pilot sites as surveillance points in China, 2010. Three surveillance points performed the oral examination and self-reported oral behavior. Respondents in Dazhu county represented the rural residents, and respondents in Nanxiong and Tianmen city represented urban residents.

Three surveillance points were selected as pilot study sites for oral health surveillance through oral examination and self-reported oral behavior, whereas other surveillance points conducted only surveillance of self-reported oral behavior. The three pilot study sites were located in Nanxiong city of Guangdong province, Dazhu county of Chongqing municipalities, and Tianmen city of Hunan province, as these surveillance points had oral examination facilities on-site and staff capable of performing oral examination (the location of three surveillance points are shown in Fig. 1). The entire sample for each point was enrolled in this study (600 subjects×3 surveillance points=1,800 subjects).

### Oral examination

According to the basic method issued by WHO oral health survey (15), gingival bleeding, calculus, and periodontal pockets were monitored using the plane mouth mirror and community periodontal index (CPI) probe, in conjunction with the probing method (probe's power <20 g) and visual examination. The dentition was divided into six sections, with index teeth 11, 16, 17, 26, 27, 31, 36, 37, 46, and 47 representing each section. Typically, testing results of index teeth represented the periodontal health for each section. All examinations were performed by uniformly trained staff. Before the survey, calibrating examiners and reproducibility of examination between inter- and intra-examiner were assessed by the *kappa* statistic. A score of *kappa* (κ) *>*0.8 indicated good agreement. If the examiner's κ score was less than 0.8, examiner would be re-trained and evaluated repeatedly till the κ score of examiner achieved more than 0.8.

### Criteria for diagnosing periodontitis

Based on the CPI value, periodontal statuses were classified into the following categories. Score 0 represented healthy periodontal tissues. Score 1 indicated gingivitis. Score 2 demonstrated calculus around periodontal tissues. Score 3 indicated 4–5 mm deep periodontal pocket which was considered as moderate periodontitis. Score 4 represented more than 6 mm deep periodontal pocket which was determined as severe periodontitis.

### Predictors

The demographic information (age, gender, educational level, annual family income, and residence location) and chronic diseases status (DM, hypertension, dyslipidemia, and obesity) were obtained from the existing 2010 CCDRFS data, in which diagnostic criteria for DM and obesity were in line with the WHO standards issued in 1999. The WHO diagnostic criteria for diabetes should be maintained – fasting plasma glucose ≥7.0 mmol/L (126 mg/dL) or 2 h plasma glucose ≥11.1 mmol/L (200 mg/dL) (16). A person with a BMI ≥30 is generally considered obese. Chinese guidelines for management of hypertension in 2010 (17) and 2007 Chinese guidelines on prevention and treatment of dyslipidemia in adults (18) were respective diagnostic criteria for hypertension and dyslipidemia in this study. Hypertension is defined as the condition in which systolic blood pressure is ≥140 mmHg and/or diastolic blood pressure is ≥90 mmHg in subjects who are not taking antihypertensive medication. Meantime, if person has hypertension history,even his blood pressures are in the normal range due to taking the anti-hypertension medication, hypertension is still be diagnosed. Total cholesterol (TC) ≥6.22 mmol/L, lipoprotein cholesterol (LDL-C) ≥4.14 mmol/L, high-density lipoprotein (HDL-C) <1.04 mmol/L, or triglyceride (TG) ≥2.26 mmol/L indicates dyslipidemia.

### Statistical analysis

We calculated prevalence estimates and 95% confidence intervals (CIs) of prevalence. Age groups were stratified into 18–44 years, 45–64 years, and ≥65 years. Education levels were classified into illiterate or primary school (≤6 years) and junior high school or higher (>6 years); annual family income factors were divided into <1,999, 2,000–3,999, and ≥4,000 USD groups. According to the diagnostic criteria for chronic diseases, diabetes, hypertension, and dyslipidemia were categorized as bivariables. We determined the characteristics of the study sample and subsequently estimated the prevalence of potential predictors. We included diabetes, hypertension, dyslipidemia, and covariate factors as categorical variables in the multivariate logistic regression model to estimate the prevalence of odds ratios (ORs) of prevalence of severe periodontitis. Data were analyzed using *SPSS* software for Windows version 17.0. For all analyses, we considered only *p* values of <0.05 significance.

The 2010 CCDRFS and this study were approved by the ethics committee of the Chinese Center for Disease Control and Prevention.

## Results

### The characteristics of study population

A total of 1,800 subjects were enrolled into this study. Of these, 1,728 subjects (96%) filled up questionnaires and completed the oral examination. The characteristics of the study population are shown in Table 1. Subjects aged 45–64 years and ones with annual family income <1999 USD respectively accounted for 53% and 46.9% of all subjects. Approximately half of the subjects were male (47.6%) and living in rural areas (53.5%). More than half of the subjects (57.6%) were illiterate or had not completed primary school. Of the four main chronic non-communicable diseases, about 55 and 45% of subjects had dyslipidemia and hypertension, respectively. The prevalence of DM was 10%, and only 3.3% of subjects were obese.

**Table 1:** The characteristics of the study subjects (*N*=1,728) in the pilot study in China, 2010

Characteristic	Number	Proportion (%)
Age group (years)		
18–44	575	33.3
45–64	915	53.0
≥65	238	13.8
Gender		
Male	823	47.6
Female	905	52.4
Residence location		
Urban	804	46.5
Rural	924	53.5
Education groups		
≤6 years	995	57.6
>6 years	733	42.4
Annual family income(USD)		
≤1,999	811	46.9
2,000–3,999	487	28.2
≥4,000	430	24.9
Hypertension	780	45.1
Diabetes mellitus	178	10.3
Hyperlipidedmia	950	55.0
BMI group (kg/m^2^)		
<18.5 (underweight)	52	3.0
18.5–24.9 (normal)	1,100	63.7
≥25–29.9 (over weight)	519	30
≥30 (obesity)	57	3.3

### The status of periodontitis

Based on the personal maximum CPI, only 10% of the subjects had healthy periodontal tissue, and 62% of the subjects had calculus. A total of 32 subjects were diagnosed with severe periodontitis according to the criteria recommended by WHO; therefore, the prevalence of severe periodontitis was 1.9% (95% CI=1.2–2.5%) (Table 2).

**Table 2:** The prevalence of periodontal status among adults in the pilot study in China, 2010 (*n*=1,728)

Personal Max CPI (Scores)	Number	Prevalence (%)	95% CI
Healthy periodontal status (0)	179	10	8.6–11
Gingival bleeding (1)	33	1.9	1.2–2.5
Calculus (2)	1065	62	59.2–64.2
Shallow periodontal pocket (3)	419	24	22.2–26.2
Deep periodontal pocket (4)	32	1.9	1.2–2.5

## The logistic regression analysis of periodontitis-related predictors

In the univariate analysis, the potential predictors for severe periodontitis demonstrated that DM, being illiterate or not having completed primary school (≤6 years), rural residence, and being male were related to severe periodontitis. However, factors such as hypertension, dyslipidemia, obesity, and annual family income were not potential predictors for severe periodontitis (95% CI of OR value included 1). In the multivariate analysis, being male was 3.5 times more likely to be a factor for having severe periodontitis than being female (OR=3.5, 95% CI=1.6–7.7).The odds of severe periodontitis was significantly higher for subjects living in rural areas (OR=2.1, 95% CI=1.0–4.9) than those in urban areas. Subjects with diabetes were 2.4 times more likely to have severe periodontitis (OR=2.4, 95% CI=1.1–5.6). Having attended junior high school or higher (>6 years) was a protective factor for getting severe periodontitis (OR=0.3, 95% CI=0.1–0.8) (Table 3).

**Table 3:** The prevalence and odds ratios of predictors for severe periodontitis in the pilot study in China, 2010

Predictor factors (*n*)	Number (*n*)/total (*N*)	Prevalence (%)	Crude odds ratios (95% CI)	Adjusted odds ratios (95% CI)	*p*
Education >6 years	7/733	1.0	0.4 (0.2–0.9)	0.3 (0.1–0.8)	<0.05
Gender (male)	22/823	2.7	2.5 (1.2–5.2)	3.5 (1.6–7.7)	
Location (rural)	24/924	2.6	2.7 (1.2–5.9)	2.1 (1.0–4.9)	
Diabetes (yes)	8/178	4.5	3.0 (1.3–6.8)	2.4 (1.1–5.6)	
Age groups (years)					
18–44	7/575	1.2	1	1	>0.05
45–64	19/915	2.1	1.7 (0.7–4.1)	1.1 (0.4–2.7)	
≥65	6/238	2.1	2.1 (0.7–6.3)	1.0 (0.3–3.2)	
Hypertension (yes)	13/780	1.7	0.8 (0.4–1.7)	–	>0.05
Dyslipidemia (yes)	23/924	2.4	2.1 (1.0–4.6)	–	
Obesity (≥30)	0/57	–	–	–	
Annual family income					
≤1,999	20/791	2.5	1	–	>0.05
2,000–3,999	8/479	1.6	0.7 (0.3–1.5)	–	
≥4,000	4/426	0.9	0.4 (0.1–1.1)	–	

## Discussion

The results of this study showed that unhealthy periodontal status is common, but the prevalence of severe periodontitis was found at low level among adults in China. The results of this study can be likened to that of another study (19), which indicated the prevalence of dental calculus, indicating poor oral hygiene among the Chinese. It proves that there is a direct relationship between oral hygiene and periodontitis. However, traditional assessments of periodontitis were focused on destruction of the periodontal attachment, in terms of attachment loss and probing pocket depths, with no consideration being given to the morphological changes of the dentition, such as plaque, gingival bleeding, and calculus, from which the periodontal attachment is being lost. Oral hygiene or lack of it among Asians shows a high prevalence of such features, which adds a further dimension to the consideration of periodontitis among China's adult population. Oral health education and oral health behavior guidance should focus on getting rid of such morphological changes by guiding people to correctly brush their teeth and use the teeth cleaning instrument or visit an oral hygienist to remove dental calculus every six months or every year. The CPI is practical and suitable for oral health survey with large size samples. Vettore et al. after comparing the examination results of CPI teeth and full mouth protocols indicated that using CPI teeth may underestimate the prevalence but overestimate the severity of periodontal status (20).

We found that the prevalence of severe periodontitis is more serious in in rural areas than in urban areas. This finding is consistent with that reported in the third epidemiological survey of national oral health in China (21). Oral examination and treatment are expensive and are not covered by health insurance in China. Moreover, the majority of rural residents do not opt for general medical insurance. Also, the oral health promotion program has not been carried out nationwide. The results of self-reported oral health behavior surveillance in 2010 CCDRFS (22) indicated that rural residents had low level of knowledge and poor oral health behavior. There was also a dearth of basic oral health care facilities and professional dentists at the community level in rural areas. These findings indicate that poor oral hygiene is a bigger problem among rural residents, and results in a higher prevalence of severe periodontitis, than among urban residents.

DM and periodontitis are common multigenetic and multifactorial chronic diseases with higher incidence with increasing age. Both these morbidities negatively affect periodontal health and systemic health, thus affecting the quality of life (23). An abundance of recent evidence has consolidated a bidirectional correlation between DM and periodontal diseases. While DM is an independent risk factor for periodontal diseases (10), periodontitis as a chronic inflammation has a negative impact on the metabolic control of diabetes (24). In particular, periodontitis ranks sixth among all complications of diabetes. This study examines the association between chronic diseases such as hypertension, DM, hyperlipidemia, obesity, and severe periodontitis among population-based sample of adults. In this study, the estimated prevalence of DM was 10.3% which is similar to the results of CCDRFS among a representative sample of Chinese adults (25). Moreover, prevalence of severe periodontitis among subjects with DM was much higher than those subjects without DM. According to the results of a published study (25), the prevalence of prediabetes was 50.1%. In the near future, diabetes will be a very important public health problem in China. As a consequence, periodontal diseases will afflict many more of the diabetic population and lead to poor quality of life if proper strategy is not envisaged to control and prevent periodontal diseases. At present, the control and prevention of five complications of diabetes are sufficiently mentioned in China Guideline for Type 2 Diabetes. Based on the results of this study, the China guidelines for Type 2 Diabetes should pay more attention to the control and prevention of periodontal disease among diabetes.

As in other studies (25–28), our study also found that attending junior high school or higher education (≥6 years) was inversely associated with severe periodontitis. Those with higher education levels are usually employed, tend to have more income, and use oral health services more. People with higher incomes are more likely to have insurance cover for dental problems and regularly visit clinics for oral examination (26). Literate people were more inclined to receive oral health education through leaflets or brochures. In contrast, people with low income have financial barriers to access oral health care; are less likely to be aware of the need for comprehensive, ongoing dental care; and are more likely to use tobacco and have a poor lifestyle (27). Conversely, lower education results in lack of adequate oral health knowledge, insufficient preventive behaviors, and low use of oral health services (28). Some have argued that the extent of access to dental care reflects most of the socioeconomic disparities in oral health (29).

In addition, in this study, males were more likely to have severe periodontitis than females, which is consistent with a recently published article (30). The composite effect of sex-specific genetic architecture and the circulating levels of sex-steroid hormones closely parallels differences in the immune response and may account for corresponding sex-related differences in risk for chronic periodontitis, with men exhibiting greater susceptibility than women (31). Furthermore, smoking is an established risk factor for poor oral health (32). Cigarette smokers are more likely to have more missing teeth and to experience greater rates of tooth loss than nonsmokers (33, 34). In 2004, the US Surgeon General reported that sufficient evidence exists to infer a causal relationship between smoking and periodontitis (33). Several hypothesized mechanisms underlie the relationship between smoking and oral health, including impairment of the immune system, alteration of the bacterial environment, increase of endodontic diseases, and decrease of salivary function (34). In China, majority of smokers are male. This might explain the higher prevalence of severe periodontitis among males than females. However, in this study, we did not collect data on tobacco use factors to confirm this deduction.

Some limitations should be noted. First, due to the low number of cases with severe periodontitis, the prevalence was not separately analyzed for the three counties or provinces. Therefore, we do not have an exact geographical distribution of periodontitis in China. Second, in this pilot study, the examination of loss of attachment was not performed to assess the destruction of periodontal attachment. Moreover, the highest scores for CPI and loss of attachment may not necessarily be found on the same tooth. Periodontitis is the assessment of current (untreated) disease surrounding the soft tissue and alveolar bone. Without the measurement of attachment loss, the results of CPI may grossly underestimate the prevalence of periodontitis. Third, this is a pilot study; therefore, the data of study may not represent the prevalence of severe periodontitis at the national level. Although age was not a significant predictor for severe periodontitis in this study, various studies have reported that the risk of periodontitis increases with age (1, 4, 5). The explanation could be that there were fewer cases of severe periodontitis aged older than 65 years old in this study. Finally, predictors of periodontitis that have been reported in previous studies, such as inadequate oral hygiene, nutrition, stress, lifestyle factors (such as alcohol consumption, tobacco use, and physical activity), were not focused on in this study.

Our study has several strengths. First, although various studies (35–37) have examined the relationship between potential predictors and oral health, they have only examined the association between a few predictors and oral health. Our study focused on a broad range of health-related risk factors related to severe periodontitis. Second, according to the results and the high response rate of this pilot study, researchers and government officials admitted to the feasibility of combining oral examination with the CCDRFS Survey in China. As a consequence, oral examination and self-reported oral health behavior were expanded to 60 surveillance points in the National CCDRFS Survey in 2013.

Our findings suggest that targeting interventions at high-risk groups may be the most vital strategy to control and prevent periodontitis. Control measures should be particularly emphasized in high-risk groups such as those with low education, diabetes, men, and rural residents. Oral health education should be promoted in order to increase the awareness of the interactive impacts of periodontitis and chronic diseases and the maintenance of good oral health. Public health strategy should support and increase access to preventive oral care through dental clinics or the community, especially for high-risk populations.
